# Biological Time Series Analysis Using a Context Free Language: Applicability to Pulsatile Hormone Data

**DOI:** 10.1371/journal.pone.0104087

**Published:** 2014-09-03

**Authors:** Dennis A. Dean, Gail K. Adler, David P. Nguyen, Elizabeth B. Klerman

**Affiliations:** 1 Division of Sleep and Circadian Disorders, Brigham and Women's Hospital, Boston, Massachusetts, United States of America; 2 Neuroscience Statistical Research Laboratory, Massachusetts Institute of Technology, Cambridge, Massachusetts, United States of America; 3 Biomedical Engineering and Biotechnology Program, University of Massachusetts, Lowell, Massachusetts, United States of America; 4 Harvard Medical School, Boston, Massachusetts, United States of America; 5 Division of Endocrinology, Diabetes and Hypertension, Brigham and Women's Hospital, Boston, Massachusetts, United States of America; 6 National Institute on Aging, National Institutes of Health, Baltimore, Maryland, United States of America; Rutgers University, United States of America

## Abstract

We present a novel approach for analyzing biological time-series data using a context-free language (CFL) representation that allows the extraction and quantification of important features from the time-series. This representation results in Hierarchically AdaPtive (HAP) analysis, a suite of multiple complementary techniques that enable rapid analysis of data and does not require the user to set parameters. HAP analysis generates hierarchically organized parameter distributions that allow multi-scale components of the time-series to be quantified and includes a data analysis pipeline that applies recursive analyses to generate hierarchically organized results that extend traditional outcome measures such as pharmacokinetics and inter-pulse interval. Pulsicons, a novel text-based time-series representation also derived from the CFL approach, are introduced as an objective qualitative comparison nomenclature. We apply HAP to the analysis of 24 hours of frequently sampled pulsatile cortisol hormone data, which has known analysis challenges, from 14 healthy women. HAP analysis generated results in seconds and produced dozens of figures for each participant. The results quantify the observed qualitative features of cortisol data as a series of pulse clusters, each consisting of one or more embedded pulses, and identify two ultradian phenotypes in this dataset. HAP analysis is designed to be robust to individual differences and to missing data and may be applied to other pulsatile hormones. Future work can extend HAP analysis to other time-series data types, including oscillatory and other periodic physiological signals.

## Introduction

Extracting physiologically relevant features and understanding the control underlying biological time-series data is challenging due to potential multiple time-scale components, non-linear relationships among physiological measures, and feed-forward/feed-back control of the system [1]. Analyzing cortisol pulsatility is challenging due to multiple endocrine systems each with its own regulatory mechanisms including circadian modulation and cortisol feedback on the hypothalamus and pituitary gland, [2]–[4]. Additional analysis challenges include hormone assay error, limitations in hormone sampling rate and individual biological variations. Pulse identification and physiologically based modeling techniques have been developed to quantify cortisol pharmacokinetic parameters and to test plausible physiological mechanisms controlling cortisol pulsatility [5]–[25]. Limitations to these analysis techniques include the need for the user to choose parameters before beginning analysis, the presence of simplifying assumptions that may not be appropriate for the data set (e.g. homogenous compartments), and the need for different methods for different hormones. Our proposed novel analysis is presented to overcome these limitations.

In addition, our method is designed to select and quantify key qualitative features originally identified during visual inspection of frequently-sampled cortisol hormone time-series data. These qualitative features include differences in pulse frequency and amplitude during sleep and wake, and inter-individual differences in circadian variation in cortisol pulse amplitude. A motivating observation was that these qualitative differences could be explained by the concept of hierarchically organized rises and falls in the data independent of cause (e.g. sleep-wake state, assay error and difference in the signal to secrete). The goal was to develop a technique that required a minimum number of assumptions and that could be applied across a wide number of conditions including different populations including both healthy controls and individuals with altered hormone concentration profiles.

**Figure 1:**
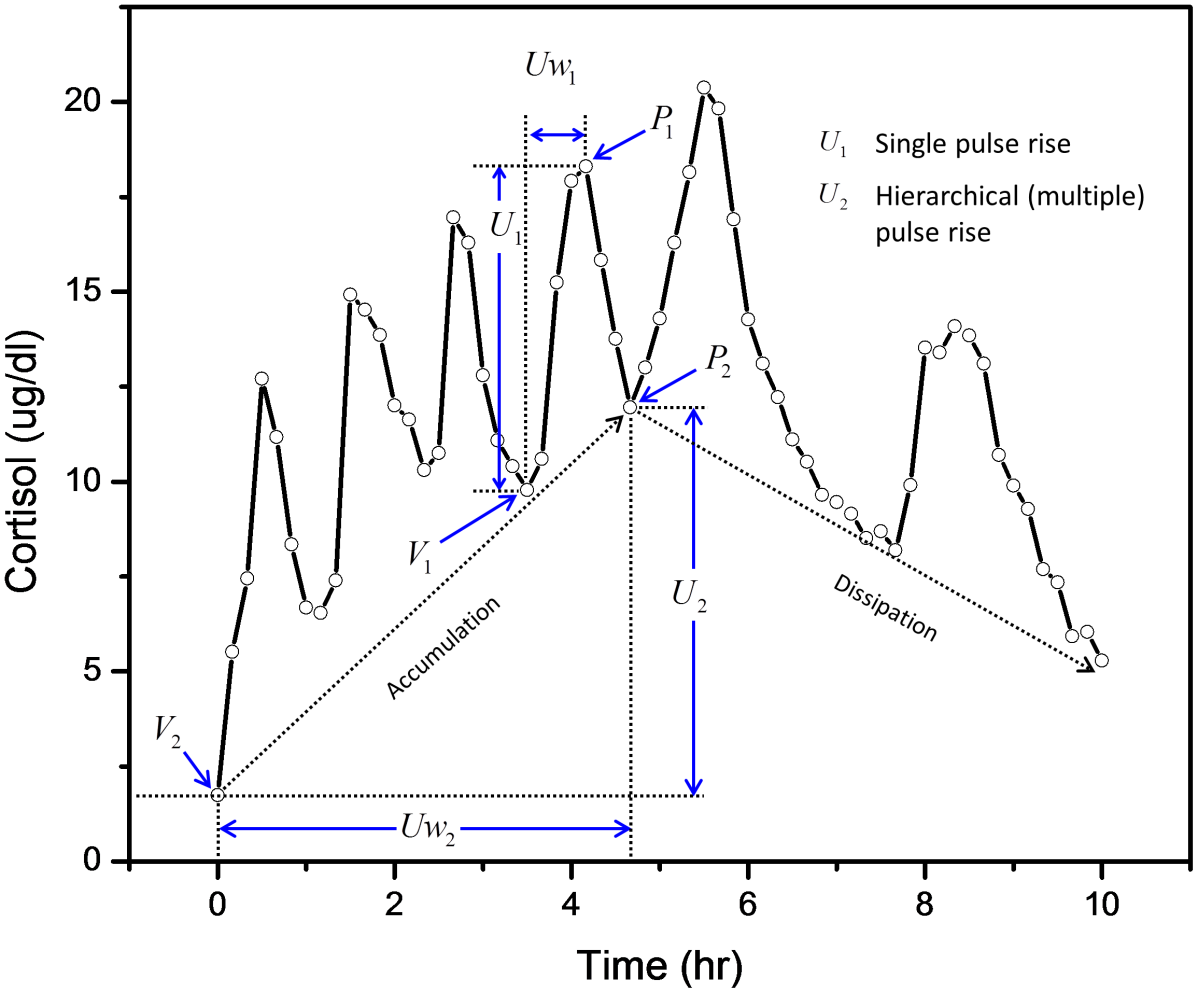
Hierarchically embedded cortisol pulses. An example adapted from experimental data (black line) with six major cortisol pulses. For a **Single Pulse Rise (U_1_)** starting at time ∼3.5 hours, the single pulse peak is identified with 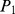 and the starting nadir/valley is identified with a V_1_. The pulse rise is defined as the rise in cortisol concentration from the nadir (V_1_) to peak (P_1_). The pulse rise time (Uw_1_) is the time required for the cortisol concentration levels to rise from a sequence of pulses starting with the local nadir (V_1_) to the peak (P_1_). The **Hierarchical (multiple) Pulse Rise (U_2_)** occurs as the change in concentration from the first nadir (V_2_) to last nadir (P_2_), which is a local maximum nadir, in the rise portion of this hierarchically organized segment. The hierarchical pulse rise time (Uw_2_) is the elapsed time between V_2_ and P_2_ of the rising portion. The effect of the hierarchical pulses between V_2_ and P_2_ is an accumulation or increase in cortisol. Similarly, sequences of pulses associated with the dissipation or decrease in cortisol begin at time ∼4.8 hours.

We present our conceptual framework by applying it to cortisol time-series data (**Figure 1**). In this paper, we demonstrate that HAP analysis of cortisol data (a) identifies salient events in multiple time-scales (e.g., pulses in one time-scale and trends in another), (b) quantifies accumulation and dissipation rates, some of which correspond to standard pharmacokinetic parameters, and (c) characterizes a hierarchical organization of the data. Although HAP is motivated by cortisol concentration profiles, the analysis structure is general enough to apply to other signals.

## Methods

### Overview

We propose a context free language (CFL) data representation [26]–[28] for curating and analyzing pulsatile biological and non-biological data. The CFL is a ‘model free’ approach and is used to identify features in the data series. CFLs are commonly used to specify the structure of programming languages and for natural language processing [29]–[31] (See **Text S1** Section A for a brief introduction, **Figure S1** for a CFL example, and **Figure S2** for a CFL string processing example). Our novel CFL framework, Hierarchically AdaPtive (HAP) analysis, converts the pulsatile time-series into a symbolic form that allows for quantitative and qualitative analyses based on novel text-based and graph-based representations of the time-series. Collectively, the tools and techniques generate rapid semi-automatic analyses of data.

A CFL-derived algorithm for data processing is defined and allows for analyses of individual and group data. The CFL is designed to capture the multi-scale features present in embedded hormone pulses: one or more secretory episodes are embedded within a longer time-scale and a larger concentration-scale rise and fall in hormone concentration values. Our CFL-based framework is extendable to oscillatory and other periodic biological and non-biological signals and can be used to quantify and understand underlying mechanisms. The rapid and semi-automatic approach within HAP analysis is also of general scientific importance due to the need for analytic tools that can be applied to the increasing size and number of data sets available.

HAP is presented as suite of integrated tools that characterize individual and group time-series. It contains more than 20,000 lines of MATLAB code that result in an analysis pipeline. One tool converts the data into an appropriate MATLAB structure to allow users to quickly use the other components of HAP. Pulsicons, a signal text representation, provides a framework for pattern analysis; the hierarchical aspect of the pulsicons allows identification of embedded patterns. Lastly, the analysis pipeline is developed with modern software techniques that include versioning and testing of core routines. For example, core routines are written as classes. Using classes is an approach to writing software that allows the core methods to be modified quickly. These software development techniques are recommended by United States Department of Health and Human Services for computer systems used in clinical investigation [32]. Thus, these software tools are structured to enable extensions to include existing and new analysis techniques within the analysis pipeline. HAP is also designed to be complementary to existing data analysis and modeling techniques. For example, its outputs can be used as input to other methods.

### Analytical Methods –HAP Analysis

**Figure 2:**
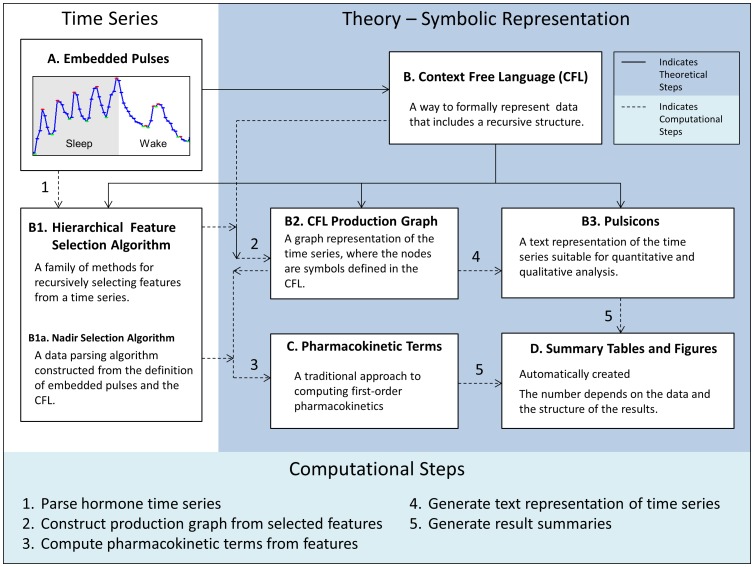
Hierarchically AdaPtive (HAP) Analysis Schematic. The figure is divided into 3 sections: Time-series (left panel), Theory – Symbolic Representation (right panel) and Computational Steps (bottom panel). Solid arrows indicate theoretical steps; dotted lines with numbers indicate computational steps listed in the bottom panel.

HAP analysis (**Figure 2**) is an analysis framework that includes theoretical and computational steps. The theoretical steps required to generate the computational steps are:

Step 1: A CFL is defined to represent pulsatility within a time-series.
Step 2: A data parser is defined to convert the time-series into a symbolic representation.
Step 3: A production graph is generated from the symbolic representation of data.
Step 4: A text representation of the time-series (pulsicons) is generated from the production graph.
Step 5: Hierarchically organized parameters are computed from traditional equations integrated within the data parser (Domain Specific Step). Note that this is the only domain specific step; it will need to be modified for each type of data.

Details of these methods are described in the **Results** section since they are major results of the paper.

### Software

#### HAP Software

HAP analysis was implemented as a collection of software tools assembled into a computational pipeline. The components of the pipeline include: (1) a general hormone database structure for organizing hormone time-series data and experiment details including experimental protocol, participant demographics and sleep-wake state, (2) tools for applying HAP to data stored in the database, (3) tools for visualizing the hormone data contained in the database, (4) tools for visualizing the results from the HAP analysis, and (5) tools for creating summaries of individual and group data results.

The HAP analysis pipeline was written with MATLAB ver. 7.3 and contains 87 MATLAB script files. The Graphical User's Interface Development Environment (Guide) toolbox was used to create an interface for selecting, visualizing, and analyzing the hormone database structure. The HAP_Analysis program used in this analysis and the source code can be found at https://github.com/DennisDean/HapSource/releases. HAP_Analysis is a windows executable and does not require MATLAB to be installed on the computer. **Text S1** contains additional program details and a summary of figures that can be generated by the program is shown in **Figure S3**. A video of the program executing an analysis is posted on YouTube (http://youtu.be/ggJDK7alI2M).

#### Cortisol Model Simulation Software

A published mathematical model of cortisol concentration levels [33] was implemented and used to test the HAP implementation. Features of the mathematical model include randomly sampled interpulse intervals with circadian amplitude modulation. The MATLAB simulation script and a test program can be found at https://github.com/DennisDean/BrownCortisolModel. A brief description of the model and software is included in **Text S1**.

### Data

#### Experimental Data

Fourteen healthy women were studied in an inpatient protocol [34]. Blood was sampled through an indwelling intravenous catheter every ten minutes for 24 hours and later assayed for cortisol. The first eight hours consisted of a scheduled sleep period and the remaining 16-hour wake period employed a constant routine protocol during which participants remained awake in a semi-recumbent position in dim lights and were given small regularly spaced meals [35]–[37].

### Ethics Statement

All participants gave informed consent, and the experimental protocol was approved by the Partners Healthcare Institutional Review Board.

## Results

The derivation for steps 1–4 of HAP analysis are presented first, followed by their application to cortisol data.

### Theoretical Results

#### Context Free Language for Identifying Hierarchically Organized Pulses (Step 1)

The CFL was motivated by the observation that a rise or fall in the cortisol concentration profile can be due to an embedded set of pulses. This multi-scale embedded feature can be represented as a hierarchical structure. The detailed construction and steps involved in a CFL example of embedded parentheses is included in Section A of **Text S1**. The details of the CFL for HAP are presented here.

Let 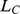 be the set of the CFL that can identify hierarchical rises and falls in a time-series representing cortisol concentration measurements. 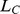 is composed of a grammar that can generate strings to represent any valid cortisol concentration or other pulsatile time-series; these strings can be transformed into a computation for recognizing the CFL directly from the pulsatile time-series.

The CFL grammar that generates these strings in the language 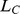 is composed of a quadruple, where a quadruple is a set of four definitions, that includes the alphabet 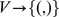, the set of terminals 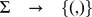, the set of rules 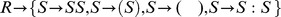, and the start symbol 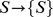. The components of the CFL grammar are shown in Equations (1)–(4).
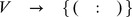
(1)

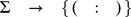
(2)

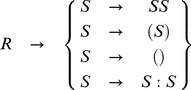
(3)

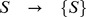
(4)


This CFL provides a symbolic and compact representation of hierarchically organized pulses. Thus, the CFL is a way to represent pulsatile time-series by discrete sections or tokens, where each section describes features present in the data as defined in the language's alphabet.

#### Nadir Selection Algorithm Specification (Step 2)

*CFL and Nadir Selection Algorithm Relationship.* The CFL described above is a compact representation of pulsatile data and can be used with time-series. However, direct application is problematic since it is not possible to deterministically identify the next valid CFL production when examining a portion of the time-series from left to right (i.e., earlier to later in time). Additional information is required to determine the next valid production according to the theory of computation [26], [28]. The nondeterministic nature or use of a push-down automata (i.e., embedded parentheses example in Section A of **Text S1**), which is the usual means of analyzing a CFL, may require more computational resources than a deterministic algorithm, and therefore computation speed can be slow. Instead the inherent structure in matched parentheses can be used to construct an efficient and deterministic algorithm for recognizing the CFL. For these pulsatile data, a deterministic partitioning algorithm is used. The idea behind our specific partitioning algorithm, the Nadir Selection Algorithm, is that processing the entire time-series simultaneously allows matching pairs of rises, peaks, and falls in the data to be determined recursively. Thus, the Nadir Selection Algorithm is a time-series parser for converting the data into a symbolic representation. In addition, since we observed that the relationship between time and amplitude of nadirs in 24 hour cortisol profiles varied substantially by individuals (details below), we conjectured that this context free representation of pulsatility could be useful as a non-parametric approach to represent the multi-scale relationship of features (e.g., nadirs and peaks) in a pulsatile time-series. Note that an equally valid algorithm anchored on peaks could have been derived. The nadirs in the time-series were selected to anchor the partitioning algorithm since hormone pulse start times are traditionally associated with nadirs.

**Figure 3:**
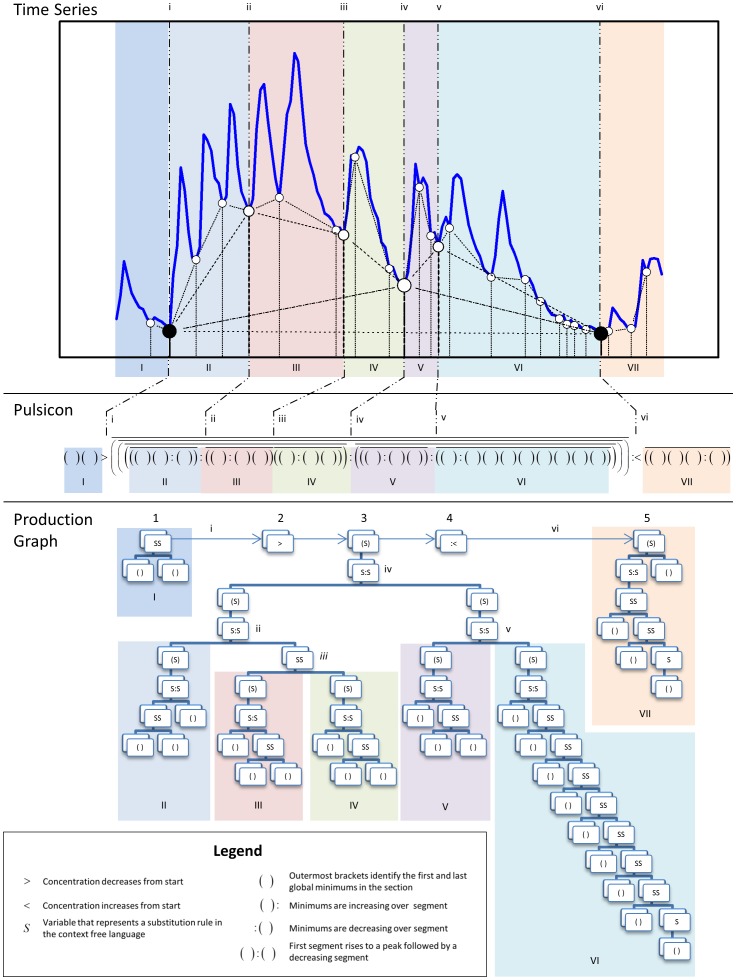
Qualitative time-series representations for a single participant (ID6). The dotted vertical lines with lower case Roman numerals represent the same time points in the Time-Series, the Pulsicon, and the Production Graph for participant ID6. Shaded rectangles labeled with uppercase roman numerals represent the same temporal interval in the Time-Series, the Pulsicon, and the Production Graph. **Time-Series:** The 24-hour cortisol time-series. Nadirs identified in each of the four HAP iterations are connected with lines. The terminal HAP nadirs are identified with black circles. **Pulsicon:** The pulsicon for the time-series. **Production Graph:** Each rectangle represents productions: the application of rules defined in the CFL The five rectangular boxes represent the key features of the time-series. The first two rectangles (Labeled 1 and 2) show that there is a decreasing portion prior to the main portion of the cortisol time-series with the “>” in rectangle 2 (e.g., before left black circle). The main portion of the cortisol time-series is represented by the center rectangle (Labeled 3) with the production symbol (S). The production symbol (S) is a variable that can be replaced with any of the rules defined in the context free language. This section contains a hierarchy of embedded pulses. The last two rectangles (Labeled 4 and 5) from the production graph show that the cortisol time-series is rising after the main portion (rectangle labeled 3) of the cortisol time-series (e.g., after right black circle).

*Nadir Selection Algorithm.* The Nadir Selection Algorithm is based on a recursive algorithm for identifying and classifying peaks and troughs within the time-series data. The first step of the algorithm is to identify local peaks and nadirs. The algorithm is recursively called with nadirs as input until no rises or falls in the data are identified (**Figure 3**). The recursion results are used to reconstruct the productions (i.e., rises and falls) required to generate the time-series. Quantitative comparisons identify relative changes within the time-series within this hierarchy of recursion results. The quantitative comparisons include expressions for identifying concentration rises and falls, plateaus, flat regions, and missing data. The recursive algorithm was designed to exploit parallelism in the data and results in an efficient method for processing data [27], [38]–[40].

Let 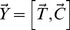 , where the time-series 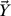 is composed of the time vector 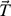 and the concentration vector 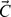. The time vector 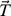 is the set of concentration sampling times 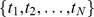, assumed to be sampled at a constant interval 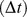, and may have missing data points. Time-based equations are presented in equations (5) and (6).
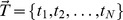
(5)

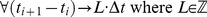
(6)Let 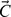 be the set of hormone concentrations values 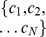 that correspond to the sampling times 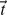 as shown in equation (7).
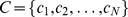
(7)


A comparator operator 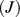 which is a function of the concentration data 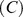 and a set of comparators 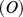 is defined in equations (8) and (9). The comparator is a tuple where each entry is a member of the set of simple comparators defined as 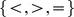. Specific pairs of operators are selected that correspond to visually relevant features of hormone concentration data relative to the center point, namely nadirs 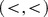, peaks 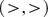, rising plateaus 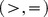, descending plateaus 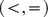, and flat regions 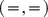. Details regarding the interpretation of the comparator operators are described in Section B of **Text S1** and the comparator operators are summarized in **Figure S4**.
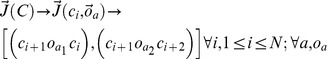
(8)

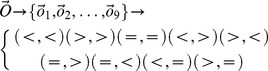
(9)


Let 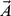 correspond to the features that are identifiable in the data as shown in equation (10):
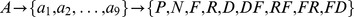
(10)where each feature 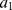 corresponds to the following one of the 9 operators described above.

Let 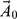 correspond to the features identified in the data by applying the comparator operator 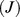 with the set of operators 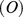 to the concentration data 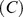.

Let 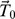 and 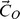 correspond respectively to the times and concentration values of the q features identified in 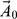. The features that identified corresponding times and corresponding concentration values are defined by equations (11)–(13).
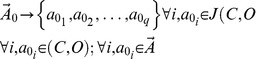
(11)

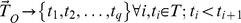
(12)

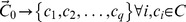
(13)


A family of recursions is defined by recursing on a subset of the identified features. A subscripted S is used to designate the selected features with corresponding times and corresponding concentration values as shown in equations (14)–(16).
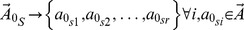
(14)

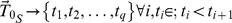
(15)

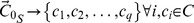
(16)


The subset 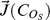 is defined to be the nadirs for the purpose of partitioning the hormone time-series into distinct pulsatile regions as described in equation (17).
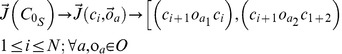
(17)


Similarly, a partitioning from peak to peak can be defined by selecting the peak as the subset.

*Algorithm Interpretation.* The identified nadirs have both a mathematical and physiological interpretation. At each level of iteration, two adjacent vertical lines in **Figure 3** identify a nadir-nadir interval with a single intervening peak; these HAP-selected nadirs identify embedded rising and falling concentration segments. Mathematically, the same concept is referred to as the forced state of the system [41]. Nadirs selected at multiple iterations are identified as fixed points in the sequential dynamical systems literature [42]–[44]. The selected nadirs correspond to the start of an increase in cortisol concentration and are likely to correspond to the approximate timing of a signal to secrete cortisol.

*Computing Accumulation Rates, Dissipation Rates, and Inter-nadir Intervals.* The extracted hierarchical features provide a concise definition of pulsatility that can be used to calculate traditional hormone pharmacokinetic measures. In this section, we present definitions of accumulation rates, dissipation rates and inter-nadir intervals derived directly from features extracted from HAP. These metrics are analogous to secretory rates, clearance rates, and interpulse intervals, respectively,

The change in cortisol concentration rate 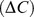 over a finite time interval 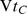 is equal to the starting concentration level 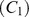 plus the cortisol secreted during the interval minus the cortisol eliminated during the interval. The cortisol generated during the interval is equal to the secretory rate 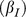 during secretion multiplied by the change in time 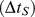. Similarly, the cortisol dissipated is equal to the clearance rate 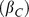 multiplied by the time cortisol is being cleared from the change in time 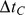. This relationship is shown in equation (18).
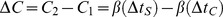
(18)


For a simple pulse, the duration of secretion is equivalent to the rise duration while duration of clearance is equivalent to the fall duration, as in equation (19).
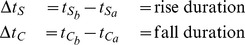
(19)

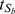
 and 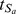 are the end and start of the rise interval, respectively. Similarly, 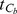 and 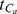 are the end and start of the fall interval, respectively. For simplicity, secretion and clearance are assumed to be temporally distinct.

Consider a subset of features from 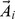 selected at iteration 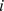 where every entry is a nadir (N), decreasing to flat (DF), or flat to rising (FR). Then there exists a single peak (P), rising to flat (RF), or flat to decreasing (FD) within 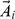 that provides sufficient information to estimate the first order secretory and clearance rates. For simplicity, consider a subset of features that contains three entries sequentially consisting of a nadir, peak, and nadir as shown in equation (20).
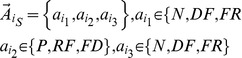
(20)


If we assume that the effective secretory period corresponds to the rising portion and the clearance period corresponds to the descending portion of the interval, then equations (11)–(13), (18), and (19) can be used to compute the first order secretory rate 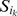, infusion rate 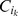, and inter-nadir interval 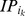 directly from HAP outputs as shown in equations (21)–(23).
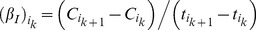
(21)

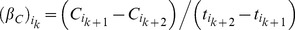
(22)

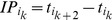
(23)


For the simple example above, there is only one secretory rate, one clearance rate, and one interpulse interval because there are no embedded pulses. For a subset of features 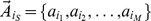 where each entry 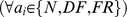, then there will be 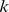 secretory rates, 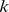 clearance rates, and 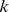 interpulse intervals where 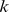 is equal to the number of local nadirs minus one. We define the hierarchical secretion rates as accumulation rates, the hierarchical clearance rates as dissipation rates and hierarchical interpulse intervals as inter-nadir intervals.

### Qualitative Time-Series Representation

The HAP analysis framework includes two visual time-series representations: production graphs (Step 3) and a text representation we term pulsicons (Step 4).

#### Qualitative Features Present in the Production Graph (Step 3)

The CFL production graph is presented as a way to describe the qualitative features present in a cortisol time-series. The CFL production graph is a summary of the steps in the language required to represent the data. The goal of the graph is to better visualize qualitative features such as the relations of embedded pulses and could be used to integrate graph-based analysis algorithms in future analytical work.

The production graph for the example in **Figure 3** is organized in a sequence of 5 rectangles that form a high level description of the time-series from left to right. Three rectangles - labeled 1, 3, and 5 in the production graph - contain the productions required to generate their corresponding pulsicon sections. These three rectangles are expanded to show the productions required to produce the pulsicon section that represents this cortisol time-series. Two rectangles (labeled 2 and 4) identify whether the data are rising or falling relative to the main section (labeled 3). Both points in time and temporal intervals are identified to reinforce the relationship between the time-series, the pulsicon, and the production graph.

#### Text Representation of a Single Time-Series (Step 4)

HAP analysis generates a pulsicon or text description of the time-series. A pulsicon is a complete record of rises and falls within the time-series and can be used to evaluate the relationship among embedded pulses. Pulsicons are composed of a set of characters 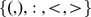. A “(” indicates the start of rise and a “)” indicates the end of a decreasing pulse. The peak between two nadirs is identified with a “:”. The “>” symbol indicates a decreasing section of the time-series and the “<” symbol indicates an increasing section of the time-series. Both “>” and “<” describe data points at either the start or the end of the time-series. A pulsicon is generated for each step in the algorithm and at each hierarchical iteration. The pulsicon generated from the production graph described above is shown in **Figure 3**.

**Figure 4:**
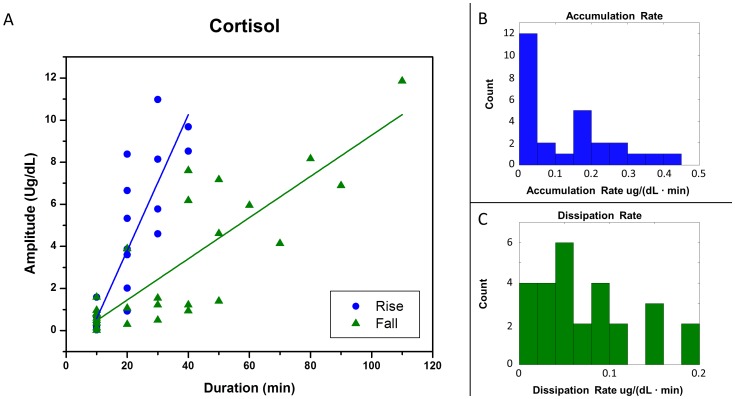
Accumulation and dissipation rates for a single participant (ID6). (**A**) Amplitude as a function of the rise or fall duration extracted from the first iteration of the HAP algorithm for participant ID6. Concentration rises (changes between local nadirs and subsequent local peaks) are shown with blue circles. Concentration falls (changes between local peaks and subsequent local nadirs) are shown with green triangles. Regression lines are shown separately for the rises (blue circles) and falls (green triangles). (**B**) A histogram of the instantaneous accumulation rates ( = rise amplitude/rise duration) from Panel A. (**C**) A histogram of the instantaneous dissipation rates ( = fall amplitude/fall duration) from Panel A.

The pulsicon text representation of a single pulsatile time-series is composed of the same alphabet used in the CFL 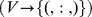 and is generated from the production graphs using standard production techniques for generating text strings from a graph representation [26]–[28]. The pulsicon provides a structure for summarizing the quantitative attributes of the time-series in a way that can be used by automated processing routines. For example, the pulsicon provides a common interface for automatically generating summary figures about the specific data set (e.g., **Figures 4** **–** **5**). Section D of **Text S1** contains (i) an additional pulsicon example and (ii) an example that demonstrates the relationship between production graphs and pulsicons (**Figure S5**).

**Figure 5:**
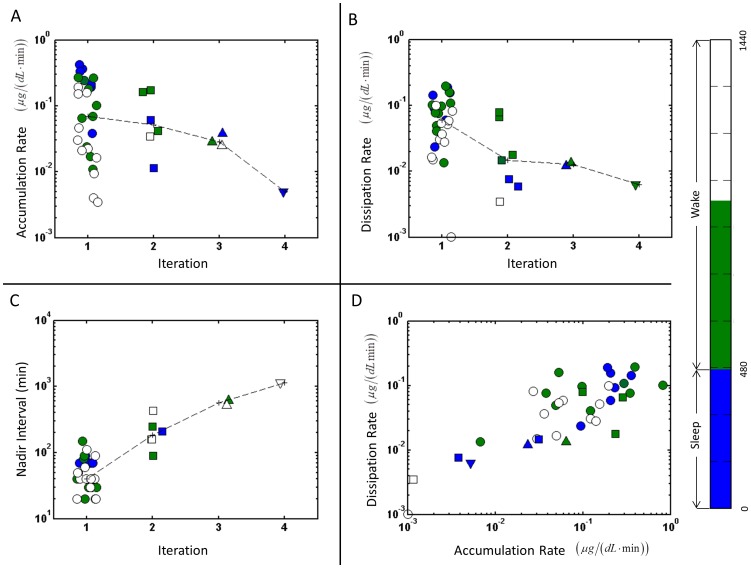
Multi-scale analysis of features identified with HAP from a single participant (ID6). Each analysis iteration for participant ID6 is shown with a different symbol: 1-circle, 2-square, 3-up triangle, 4-down triangle. The symbol color indicates the timing of the event with the color code in right-most vertical panel: the sleep episode (blue), the first 8 hours of wake (green), or second 8 hours of wake (white). The dashed lines connect median value between iterations. (**A**) Cortisol accumulation rates for nadirs identified at each iteration. Accumulation rates for the first iteration are analogous to secretory rates. (**B**) Cortisol dissipation rates for nadirs identified at each iteration. Dissipation rates for the first iteration are analogous to clearance rates. (**C**) The nadir intervals identified at each iteration are shown. The event intervals at the first iteration are analogous to the inter-pulse interval. (**D**) The relationship between an accumulation rate and the immediately following dissipation rate.

### Application of HAP analysis to simulated data

**Figure 6:**
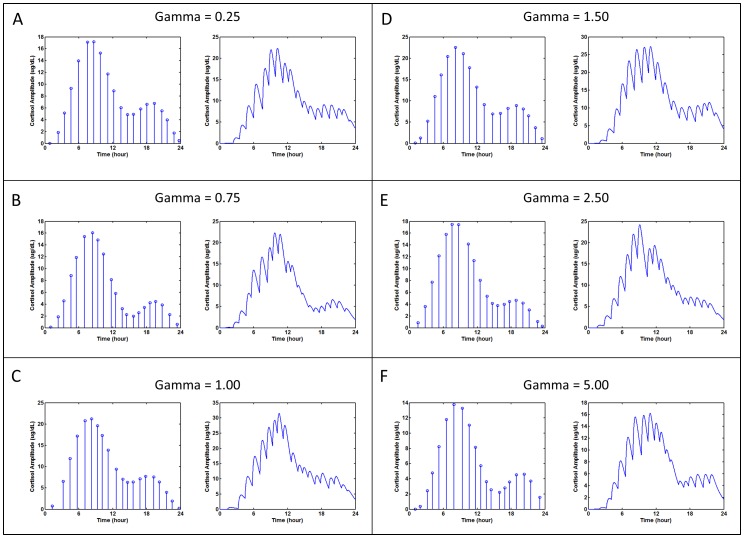
Simulated 24-hour cortisol concentration time-series profiles with gamma values from Table 1. Within each panel, the left figure plots the time and amplitudes of each pulse and the right figure plots the resulting simulated dataset. The gamma value affects the clearance rate. (A) Gamma = 0.25 (B) Gamma = 0.75 (C) Gamma = 1.0 (D) Gamma = 1.5 (E) Gamma = 2.5, (F) Gamma = 5.0.

HAP Analysis output was tested with a simulated data set for which the number, timing, and amplitude of pulses were known. The simulated cortisol test set shown in **Figure 6**. The interpulse interval and circadian amplitude variation were sampled randomly from published distributions with different gamma parameter values. The amplitudes and simulated secretion times are shown in **Table 1**. The gamma parameter is defined as a renewal process that affects the clearance of the simulated pulse. The MATLAB script files used to generate the simulated data can be found at https://github.com/DennisDean/BrownCortisolModel. A brief description the model and location for the simulation code are in Section B of **Text S1**. We used these simulated parameters to validate both the HAP analysis approach and the program implementation

**Table 1:** Amplitude (A) and secretion times (T) for simulated dataset*.

	Gamma = 0.25		Gamma = 0.75		Gamma = 1.0		Gamma = 1.5		Gamma = 2.5		Gamma = 5.0	
Pulse	A	T	A	T	A	T	A	T	A	T	A	T
ID	µg/dL	min	µg/dL	min	µg/dL	min	µg/dL	min	µg/dL	min	µg/dL	min
1	***0.02***	***51***	0.11	60	0.79	78	0.08	62	0.87	90	***0.00***	***60***
2	1.89	140	1.87	140	6.54	194	1.28	117	3.63	166	0.38	114
3	5.13	208	4.54	201	11.89	267	5.21	193	7.75	241	2.45	191
4	9.30	277	8.85	278	17.20	344	11.07	271	12.14	314	4.77	245
5	13.98	358	11.92	333	20.75	426	16.12	336	15.82	389	8.23	314
6	17.15	453	15.43	421	21.22	497	20.39	406	17.52	454	11.82	394
7	17.16	519	16.07	505	19.64	560	22.51	489	17.46	522	13.76	472
8	15.29	590	14.88	561	17.37	610	21.04	571	14.15	626	13.27	563
9	11.73	670	12.47	621	13.89	673	17.77	639	11.35	684	11.06	634
10	8.90	730	8.17	705	9.44	759	13.24	715	8.08	752	8.14	702
11	6.03	809	5.86	752	7.10	829	9.16	795	5.34	826	5.73	758
12	4.88	877	3.24	822	6.38	879	6.90	884	4.16	882	3.62	819
13	4.92	941	2.25	869	6.38	936	7.06	968	3.75	937	2.57	867
14	5.83	1017	1.96	941	7.15	1009	8.24	1048	4.01	1008	2.22	958
15	6.65	1083	2.53	998	7.76	1069	8.94	1137	4.44	1065	2.81	1013
16	6.79	1163	3.44	1057	7.58	1157	8.10	1209	4.68	1133	3.59	1064
17	5.53	1243	4.23	1117	6.45	1215	6.49	1265	4.20	1206	4.52	1137
18	4.00	1298	4.48	1176	3.99	1292	3.67	1338	3.03	1273	4.62	1220
19	1.76	1371	3.88	1246	1.92	1352	1.14	1409	1.10	1361	3.72	1288
20	0.51	1424	2.26	1327	***0.22***	***1424***			***0.27***	***1412***	1.58	1385
21			0.60	1406								

Bolded entries were not identified by HAP Analysis.

#### HAP_Analysis Verification

**Figure 7:**
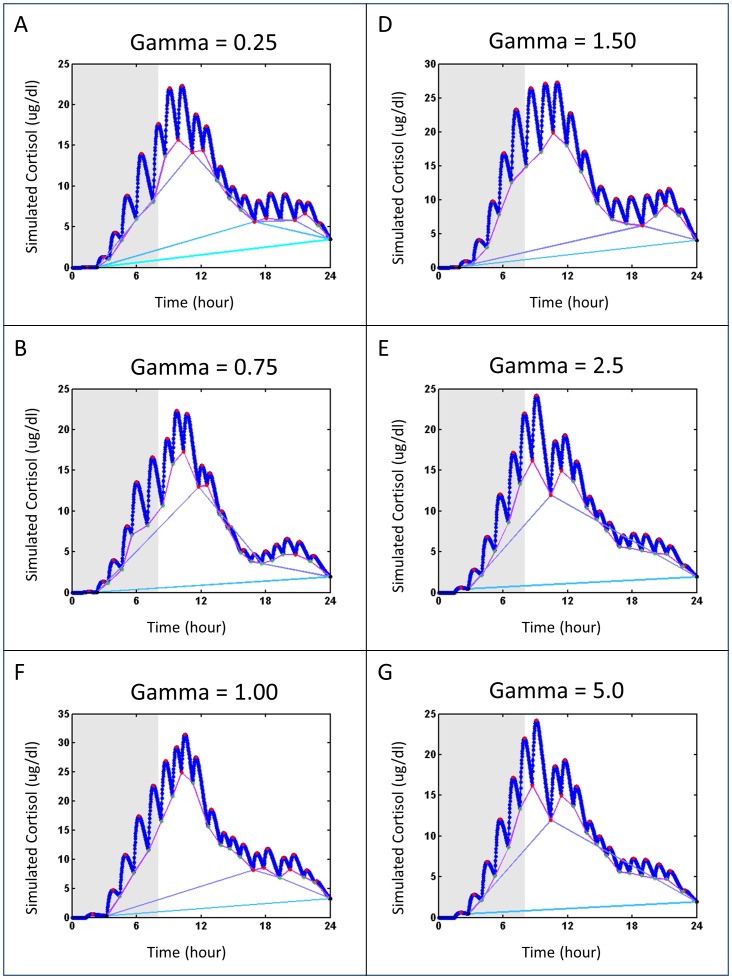
HAP analysis of simulated cortisol test set. (A–F) Simulated data generated from the data sets shown in Fig. 4 panels A–F. Simulated data are identified with blue markers. Peaks identified during the nadir selection algorithm are in red. Nadirs identified at a given recursive step are joined with a line, where line color represents a different recursive step.

HAP analysis converged for each simulated data set. The program identified rise time and inter-pulse intervals for 97% percent of the pulses (116 of 120, **Table 1** **,** **Figure 7**). The pulses missed by the algorithm had secretion amplitudes of less than 0.27 ug/dL. The amplitude of the missed pulses was not sufficient to impact the simulated time series profile. We would not expect HAP to identify these low amplitude events.

### Application of HAP analysis to experimental cortisol data (Step 5)

Within seconds HAP generated individualized accumulation and dissipation rates for all participants. **Tables 2** and **3** show results for all participants; single participant results are shown in **Table 4** and **Figures 4** **–** **5**. The Nadir Selection Algorithm parsed the data for all participants in either 3 or 4 recursive steps (**Table 2**). In addition, the software pipeline automatically created graphs, summary tables, and text descriptions of each cortisol profile in minutes.

**Table 2:** Pulsicons for All Participants.

Pulsicon by Iteration Level				
ID	1	2	3	4
**1**	(((()()():())(():()()):(():())(():()()()()())(():()())(():())(():()()()()())))	((()():()()()()()))	(())	
**2**	(((()()():())(()()():()()):(():()()()())(()():()()()()()()())(():())))	((()():()()()))	(())	
**4**	(((():())(()():()()):(()()()()():()())(():()())(():())(():()()())(():()()())))	((()():()()()()()))	(())	
**5**	(((()()()()()():()):(()():()()()()()()())(()()()():()()()())(():()()()())))	((():()()()))	(())	
**6**	(((()()()()()():())(():()()()):(()()()():()()())(():()()()()()())))	((()():()()))	(())	
**8**	(((()():())(()():()):(():()()()()())(():()())(():()()()()()()()())(():())))	((()():()()()()))	(())	
**11**	(((():())(()():()()):(():()()()())(():()()())(()():()()()()()()()())))	((()():()()()))	(())	
**12**	(((():())(()()():()()):(():()()()())(()():()()())(():()())))	((()():()()()))	(())	
**13**	(((()():())(()():())(():()()):(():())(():()())(():()()()())))	((()()():()()()))	(())	
**3**	((((()():())(()():()())(():()()):(():())):((():()()())(()():()):(():()()))))	(((()()():()):(()():())))	((():()))	(())
**6**	((((()():()):(():()())(():()())):((():()()):(()()()()()()()()()))))	(((():()()):(():())))	((():()))	(())
**7**	((((()()()()()():())(():()()):(()():())):((():()()):(():()()()()))))	(((()():()):(():())))	((():()))	(())
**9**	((((():())(():()):(():()))((():()):(():())):((():()()()()):(():()()())(():())(()():()()()()()()))))	(((()():())(():()):(():()()())))	((()():()))	(())
**10**	((((():())(()()()():()):(():()())(():())):((()():()):(()():()())(()():()))))	(((()():()()):(():()())))	((():()))	(())
	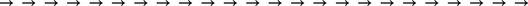			
	Simpler Time-Series Representation			

The table is organized by number of iterations: 3 for 9 participants and 4 for 5 participants. As iteration level increases, the representation appears simpler.

**Table 3:** Accumulation and dissipation rates computed from the Level 1 iteration for all participants.

	Accumulation Rates			Dissipation Rates		
Participant	Mean (95% Confidence)		R^2^	Mean (95% Confidence)		R^2^
ID	µg/(dL • min)			µg/(dL • min)		
**1**	0.18	(0.11, 0.26)	0.41	0.06	(0.03, 0.09)	0.28
**2**	0.15	(0.11, 0.20)	0.58	0.05	(0.03, 0.07)	0.49
**3**	0.14	(0.06, 0.22)	0.27	0.08	(0.04, 0.12)	0.35
**4**	0.25	(0.18, 0.32)	0.59	0.10	(0.08, 0.12)	0.64
**5**	0.24	(0.18, 0.29)	0.74	0.10	(0.06, 0.13)	0.53
**6**	0.32	(0.25, 0.39)	0.78	0.10	(0.07, 0.12)	0.71
**7**	0.21	(0.15, 0.27)	0.67	0.08	(0.05, 0.11)	0.56
**8**	0.13	(0.07, 0.19)	0.43	0.10	(0.08, 0.13)	0.73
**9**	0.17	(0.15, 0.39)	0.73	0.08	(0.05, 0.11)	0.52
**10**	0.17	(0.09, 0.26)	0.45	0.07	(0.03, 0.11)	0.39
**11**	0.27	(0.18, 0.36)	0.54	0.06	(0.03, 0.09)	0.42
**12**	0.33	(0.27, 0.39)	0.84	0.09	(0.05, 0.19)	0.54
**13**	0.07	(−0.12, 0.15)	0.14	0.05	(0.02, 0.09)	0.34
**14**	0.11	(0.09, 0.13)	0.86	0.06	(0.03, 0.09)	0.43
*mean:* 0.20 µg/(dL • min)			*mean:* 0.08 µg/(dL • min)			
*minimum:* 0.07 µg/(dL • min)			*minimum:* 0.05 µg/(dL • min)			
*maximum:* 0.33 µg/(dL • min)			*maximum:* 0.10 µg/(dL • min)			

**Table 4:** Hierarchical Features Summary by Iteration Level for a single participant (ID6).

	Iteration									
	Level 1			Level 2			Level 3			Level 4*
	Min.	Median	Max.	Min.	Median	Max.	Min.	Median	Max.	Median
Rise duration (minutes)	10	10	40	30	60	140	90	160	210	620
Fall duration (minutes)	10	30	110	40	130	400	410	420	430	520
Inter-nadir interval (minutes)	20	40	150	90	185	430	520	570	620	1140
Rise amplitude (µg/dl)	0.04	0.93	10.98	0.92	4.66	8.57	2.57	5.97	8.04	3.09
Fall amplitude fall (µg/dl)	0.01	1.22	11.86	0.14	2.5	8.57	4.96	5.38	5.81	3.24

*Level 4 is the terminal iteration for participant ID6. Since there are only two points at the terminal iteration, only the median (defined for two points as the average) is shown.

There is a difference in patterns for participant patterns with 3 vs. 4 iterations. Participants with 4 iterations have more pulses that are embedded (i.e., ( ( ) ) ) while those with 3 iterations have more simple rises and falls (i.e., ( ) ( ) ( ) ).

As an example, for participant ID6, the Nadir Selection Algorithm identifies 27 peaks and 28 nadirs from the 145 points present in the data at the first recursive step. In the second recursive step, 7 peaks and 7 nadirs were identified. In the third step of the algorithm, 3 peaks and 3 nadirs were identified. In the fourth and terminal recursive step, 1 peak and 1 nadir were identified. (**Table 4**)

#### Accumulation and Dissipation Rates

The accumulation rate and dissipation rates are calculated from the first HAP iteration (**Figure 4**) for participant ID6. The median accumulation and dissipation rates across all points are 0.067 and 0.059 µg/(dL min), respectively for this individual. Fitting a regression line to the rise points results in a 0.322 µg/(dL min) accumulation rate (R^2^ = 0.78). Fitting a regression line to the falling points results in a 0.098 µg/(dL min) dissipation rate (R^2^ = 0.71). . Fitting regression lines allows computing average accumulation and dissipation rates for the time-series. The slope of the regression is analogous to secretion and clearance rates.

#### Multi-Scale Analysis

Using equations 17–19 the accumulation rate, dissipation rate, and inter-nadir interval at each iteration for participant ID6 are shown in **Figure 5 A–C**. At each iteration, the accumulation and dissipation rates and the point spread decrease. This decrease in point deviation is due to the change in time-scale at each iteration. The fastest accumulation rates occur at the end of scheduled sleep time and many of the fastest dissipation rates occur during sleep. The dissipation-accumulation plot (**Figure 5D**) shows an approximately log-log relationship between accumulation and dissipation across alliterations. The multi-scale analysis provides a way to assess the effect of a sequence of pulses on concentration profiles.

#### Accumulation Rates and Dissipation Rates

The average accumulation rate for the 14 participants was 0.20 µg/(dL min) and ranged between 0.07 and 0.33 µg/(dL min) when all the points from the first iteration are included (**Table 3**). The R^2^ value for each participant, as computed during the linear regression of rises and falls in the data, ranged between 0.14 and 0.86. The average dissipation rate was 0.08 µg/(dL min) and ranged between 0.05 and 0.10 µg/(dL min); its R^2^ value ranged between 0.28 and 0.73. There was a positive correlation between the accumulation and dissipation rate (Pearson = 0.44).

#### Multi-Scale Analysis

HAP analyses produces results on at least two time scales: the pulse scale in the order of minutes and the ultradian scale (less than 24 hours). For the 9 participants that converged in 3 iterations, the pulse scale and ultradian scale components are, 0.67 and 2.67 hours, respectively. In the 5 participants that converged in 4 iterations, HAP computed a pulse scale and two ultradian scale components corresponding to 0.67, 2.33, and 8.17 hours. We postulate that the participants whose hormone time series converge in 3 vs. 4 iterations may have differences in cortisol control. These inter-participant differences appear at the second iteration index in the pulsicons (**Table 2**); pulse clustering (i.e., start of a new pulse before the effects of previous pulses are completely dissipated) occurs in those who converge in 4 iterations. Further details of how pulsicons represent these differences are included in **Text S1**.

## Discussion

HAP analysis demonstrates the applicability of CFLs to biological time-series data. This CFL for representing pulsatile time-series is novel and complementary to CFL methods developed for other fields [45]–[49]. HAP analysis is an objectively reproducible time domain method that efficiently characterizes the visually salient features in the data. Additional advantages are that HAP requires a minimum set of assumptions and does not require the user to set analysis parameters such as range or threshold. HAP analysis partitions the time-series temporally into hierarchically organized segments that may facilitate insight into the individual and group nature of pulsatility. The CFL provides a seamless interface for subsequent multi-scale analysis. HAP is efficient, providing individualized estimates of parameters in seconds of computing time. When applied to pulsatile time-series data, HAP provides quantitative estimates analogous to pharmacokinetic parameters and qualitative information through the use of production graphs and the novel text representation called pulsicons.

### Novel concepts introduced

#### Language-Based Pulse Analysis Technique and Analysis Pipeline

The ability to automatically describe pulsatility formally is a novel HAP analysis feature. This feature facilitates the development of an analysis pipeline, and it is intrinsically tied to visualizations. This theoretical framework for HAP is implemented as an extendable analysis pipeline whose design is motivated by automated methods derived from CFLs and current hormone analysis programs [15], [16] and with the aim of translating HAP methods into use. Our pipeline provides support for analyzing data collected in a database, which differs from many current interfaces that analyze data from one participant at a time [15], [16].

#### Hierarchically Organized Results

The hierarchically organized results from HAP analysis motivated a system of visualizations for summarizing pulsatility present in a time-series. For example, plots that illustrate the multi-scale nature of pulsatility (e.g., **Figure 5**) both provide a way to evaluate how features and parameters of the system are changing at different time-scales and provide insight into the number and organization of pulses. Visualizations for identifying the embedded nature of cortisol pulsatility (e.g., **Figure 3**) are another novel application of CFL production graphs. Pulsicons provide a new tool for understanding the pulsatility embedded within a hormone time-series through the ability to view the time-series as a text string. The pulsicons provide a framework to characterize and to potentially search time-series; this is a topic for future work. Linking HAP analysis output to visualizations is designed to facilitate verifying, validating, and analyzing results and to empower the user to explore features present in their data. The interface between generating HAP results and generating graphs is designed to employ grammar generated visualizations [50], which will allow for new visualizations to be generated interactively by the user.

### Implications of cortisol pulsatility results

The HAP analysis presented in this paper demonstrates that the approach can identify known quantitative and qualitative cortisol pulsatility features [13], [51], [52]. (i) Mortlola et. al presented pulse secretion durations and amplitudes from women that are consistent with analogous values presented in this paper [51]. (ii) HAP analysis identified the same range of ultradian components of a 24 hour cortisol time-series as reported in Veldhuis et. al [13]. (ii) Our result of cortisol concentration levels in healthy participants rising during sleep, spiking to highest levels upon wake time, and falling to lowest levels near bedtime or early in the sleep episode is also consistent with findings from other methods. This quiescent period is seen in healthy individuals, and its absence is associated with depression in women [51].These classic features of cortisol pulsatility can be identified in both the production graph and the pulsicon representation of the time-series. The quiescent period, where there are few or no pulses, in the later day is clearly identified in the production graph representation and the associated pulsicon (section VI of **Figure 3**).

### Novel HAP Generated Cortisol Findings

The qualitative and quantitative HAP analysis presented in this paper supports a complex view of cortisol pulsatility. The production graphs and pulsicons for each individual suggest a range of patterns in cortisol pulsatility over a 24-hour period that may reflect individual differences, different physiologic states (e.g., sleep or stress), pharmacology and/or response to environmental exposures. This suggests that methods for analyzing cortisol may need to adapt to individual differences present in the time-series. Specific HAP-identified findings are described below.

#### Expression of an Eight Hour Ultradian Rhythm in a Subset of Participants

HAP identified an 8-hour periodicity in the subset of participants that converged in 4 iterations and that was not present in the group that converged in 3 iterations. The approximate 0.7 and 2.5 hour signals were identified in all participants by HAP; Veldhuis identified similar periodicities in 24-hour cortisol concentration profiles [13]. However, Veldhuis did not report an 8 hour periodicity (See **Multi-scale Analysis** section in **Group Results**). We conjecture that the inter-individual difference in ultradian signal could be due to individual differences in how time-of-day affects the Hypothalamic-Pituitary-Adrenal (HPA) axis or in the effect of the 8-hour sleep episode on cortisol pulsatility. Our conjecture that sleep is implicated is supported by a recent article that reports ultradian components in rodent sleep [53].Future work including more than 24 hours of data and more than one sleep episode per person will be needed to define the characteristics of this finding.

#### Qualitative Structure of Cortisol

In this paper, we report an objectively derived qualitative structure of cortisol pulsatility that is applicable to a range of cortisol profiles and is represented visually as a production graph and text string. These qualitative structures are representation patterns of pulsatility. Since patterns of hormone pulsatility affect responsiveness to the hormone [54]–[56], it is important to understand and define the underlying patterns. We hypothesized that the qualitative structure of cortisol pulsatility reflects the effect of sleep on cortisol production and is associated with ultradian signals.

### Method Limitations

The formal pulsatility definition in this method requires an extension to the traditional definitions of pulsatility to include multiple time-scales and the pharmacokinetic measures of accumulation, dissipation, and inter-nadir interval. Because the method does not distinguish between ‘pulse counting’ and noise within the time-series, the results from the output of the first iteration may include both pulses and noise. Thus, the results from the first iteration are individualized lower and upper bound of values that include noise. Although noise, such as assay noise, may not be physiologically meaningful, the inclusion of low amplitude events within the results does have advantages. For example, the presence of a significant number of low amplitude events may suggest increased negative feedback, suppression of the firing signal, or sensitivity changes in the system's response to input, either hypothalamic or from the pituitary gland. The large quantity of low amplitude events within the cortisol data suggests that noise is an important feature of the system; a challenge is that noise characteristics may change with time of day or disease state. Traditional ‘smoothing’ of the data may eliminate or minimize noise may destroy information required to understand HPA axis control. So as to better differentiate between assay and system noise, future versions of HAP could include an assay error term or other error term suitable for the signal under study.

### Future work

Further research is required to link hierarchically organized segments with mathematical statements of relevant physiology. Linking HAP to physiological concepts through the use of observational dynamical models [12], [13], [24], [33], [57] and cellular models could provide an approach to testing relationships between HAP partitions and mechanisms of HPA control. For example, observational dynamical models could link ACTH, which stimulates cortisol release, with cortisol time-series. These models could be used to estimate higher order pharmacokinetic terms as well as to test specific hypotheses about different elements within a hierarchical control system that includes feedback as well as other elements, such as circadian rhythms or sleep-wake state, that affect their production. Application of HAP to different physiologic and pathophysiologic cortisol time-series data, including segments with and without sleep, will reveal how HAP can be used to quantitatively and qualitatively explore changes associated with these conditions [13], [58]–[68].

## Supporting Information

Figure S1 **Specification of a CFL for recognizing parenthesis.**
(TIF)

Figure S2 **Processing of a string of parenthesis with a CFL.**
(TIF)

Figure S3 **HAP_Analysis output.**
(TIF)

Figure S4 **Definition and interpretation of the comparator operator.**
(TIF)

Figure S5 **Linking time-series (data), the production graph and pulsicons.**
(TIF)

Text S1 **Supportive text and examples.**
(DOCX)

Text S2 **Includes background and additional examples.** **Table S1.** Fields description for HAP_Analysis input structure. **Table S2.** Fields description for HAP_Analysis Program Data Structure.
(DOCX)
