# Supplementary figures and images for: Biological Time Series Analysis Using a Context Free Language: Applicability to Pulsatile Hormone Data
Source: PLoS One. 2014 Sep 3;9(9):e104087. doi: 10.1371/journal.pone.0104087 (PMC4153563; doi:10.1371/journal.pone.0104087)

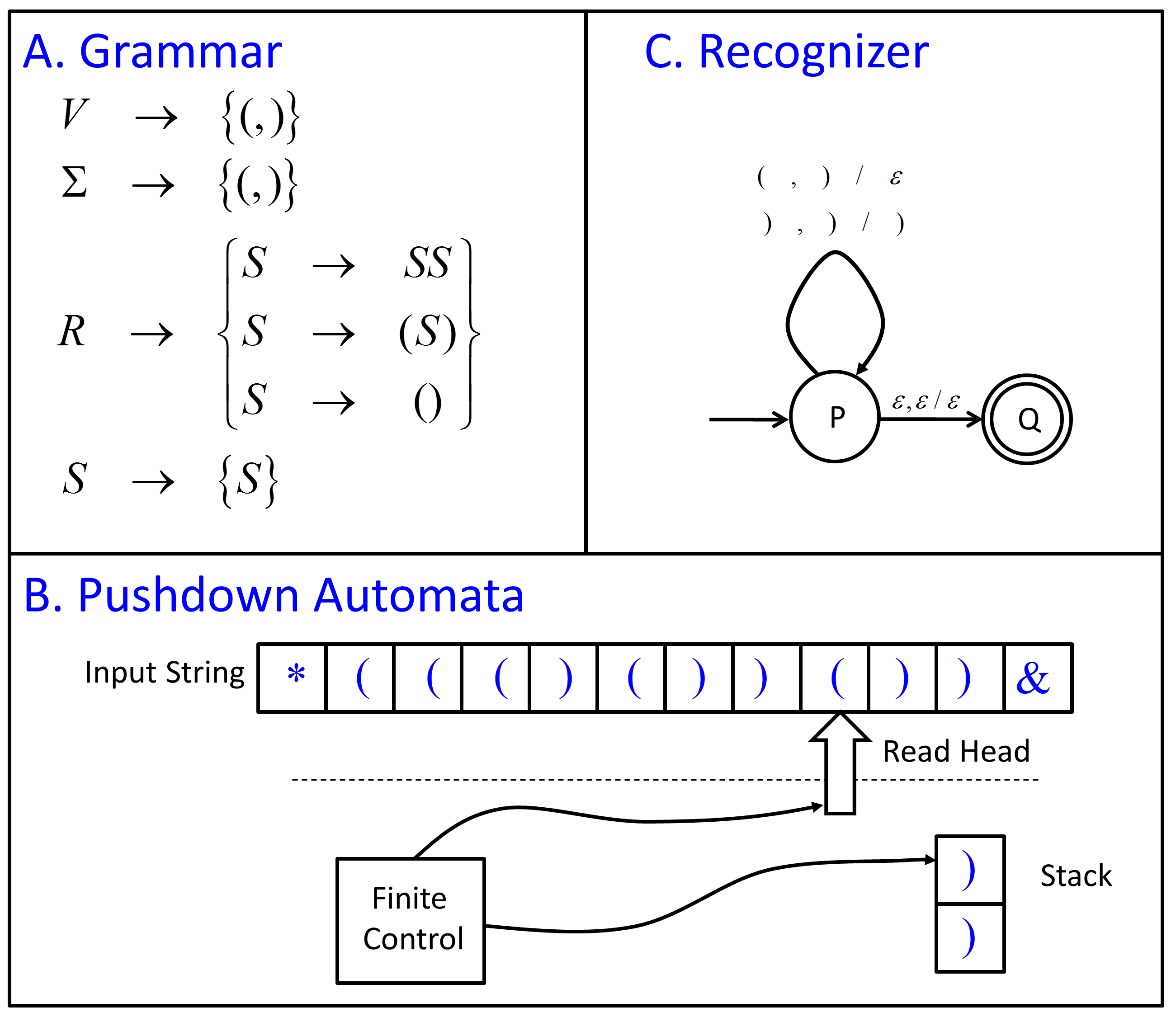

Supplement: Figure S1 — Specification of a CFL for recognizing parenthesis. (TIF) [file pone.0104087.s001.tif]

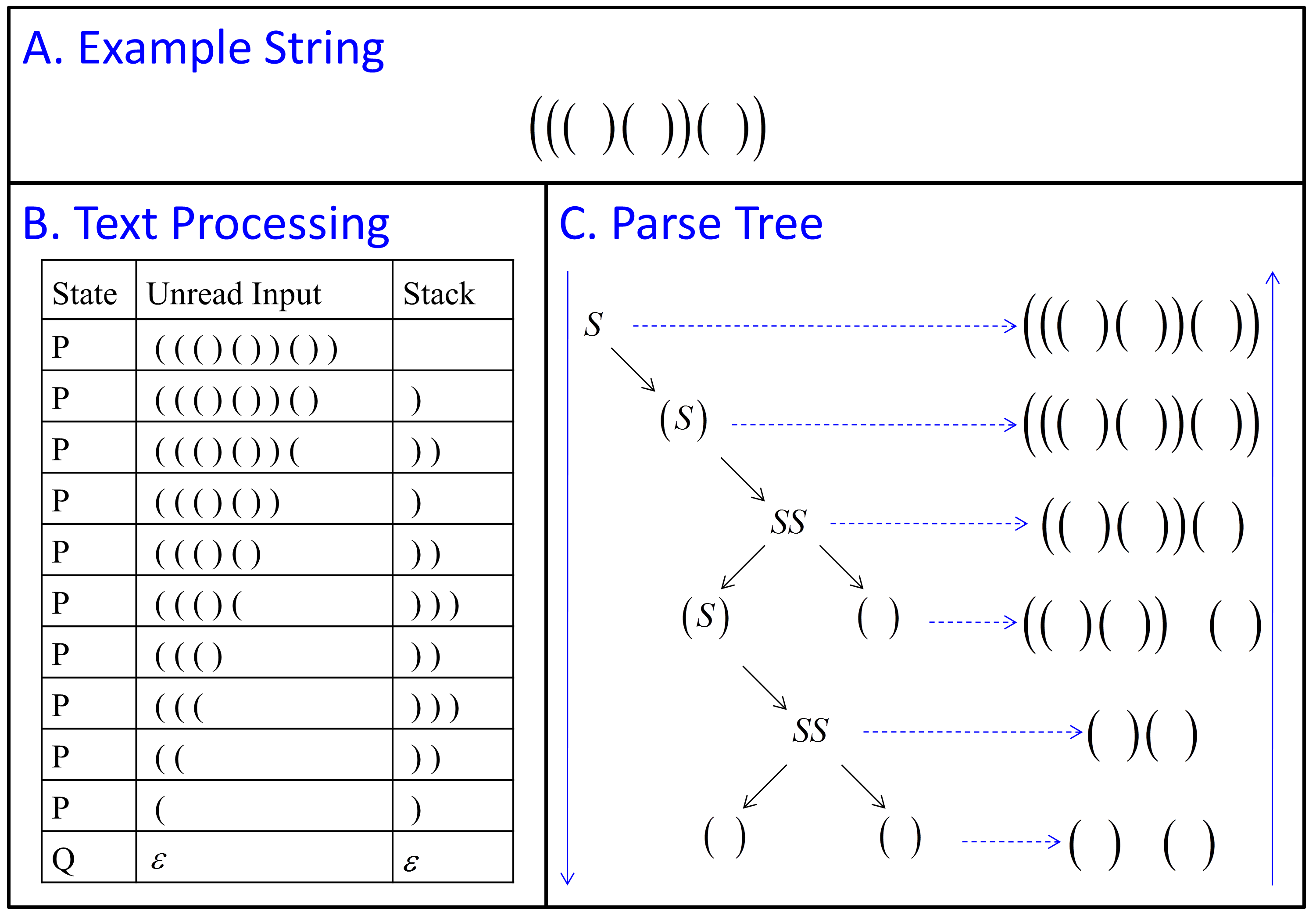

Supplement: Figure S2 — Processing of a string of parenthesis with a CFL. (TIF) [file pone.0104087.s002.tif]

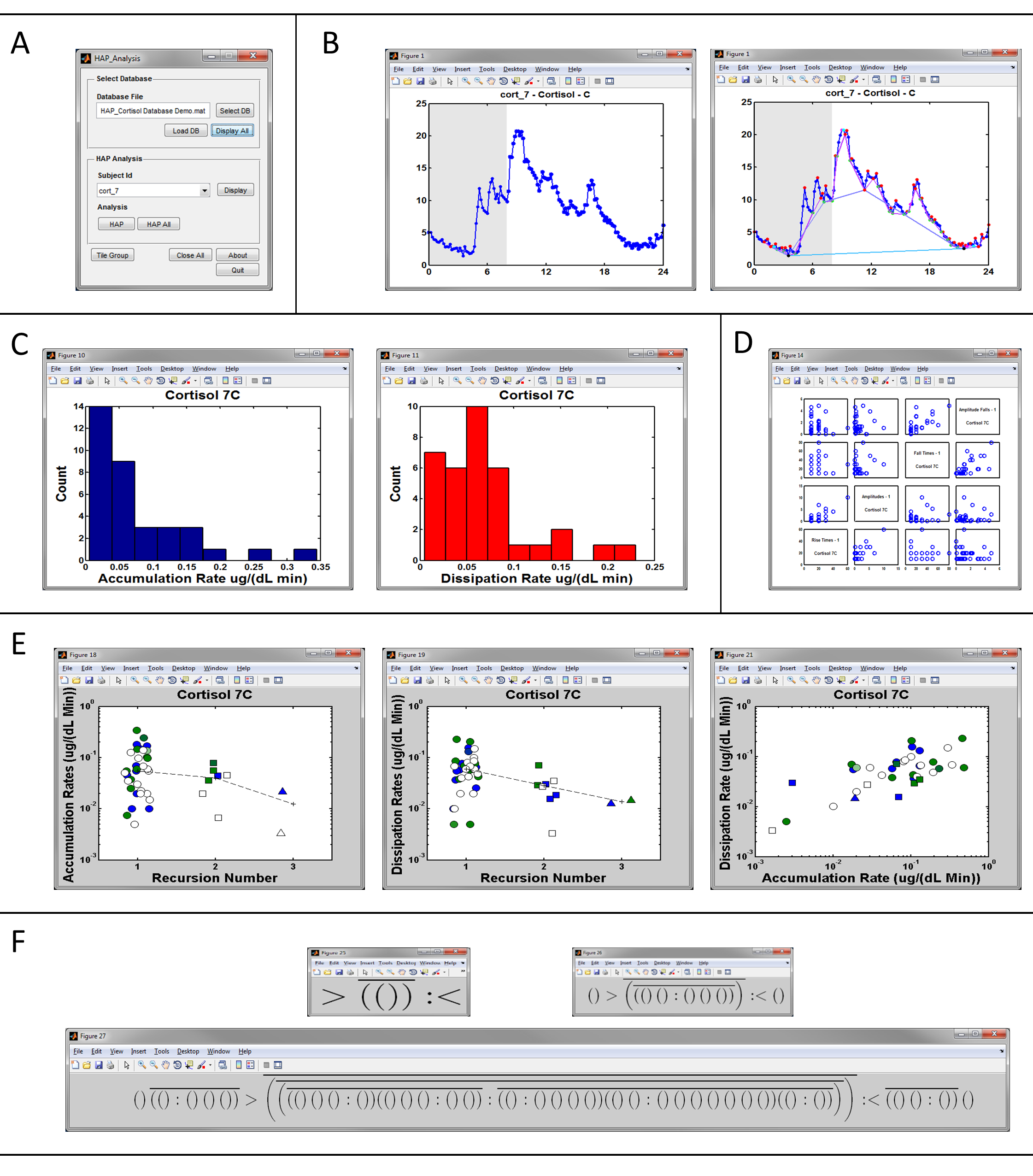

Supplement: Figure S3 — HAP_Analysis output. (TIF) [file pone.0104087.s003.tif]

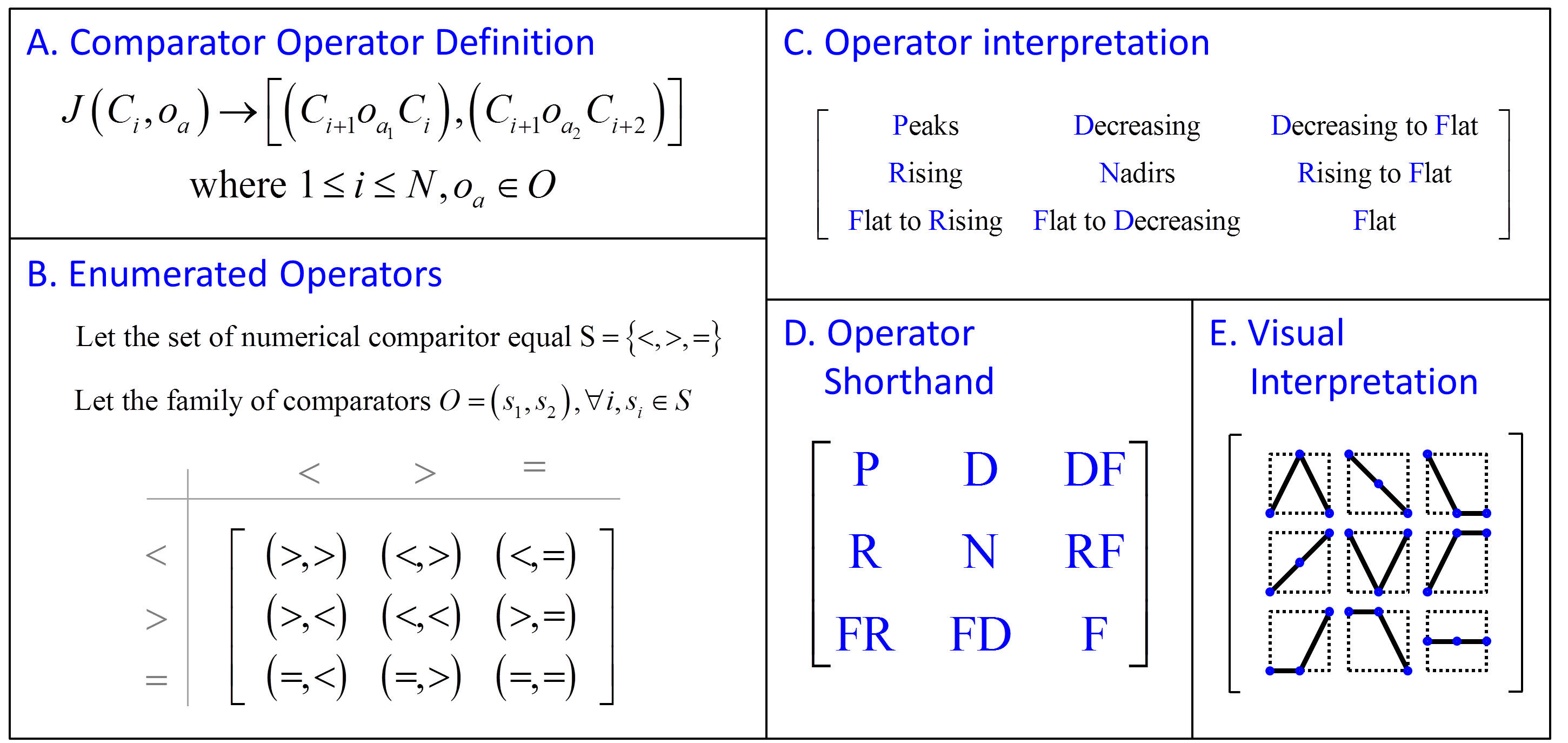

Supplement: Figure S4 — Definition and interpretation of the comparator operator. (TIF) [file pone.0104087.s004.tif]

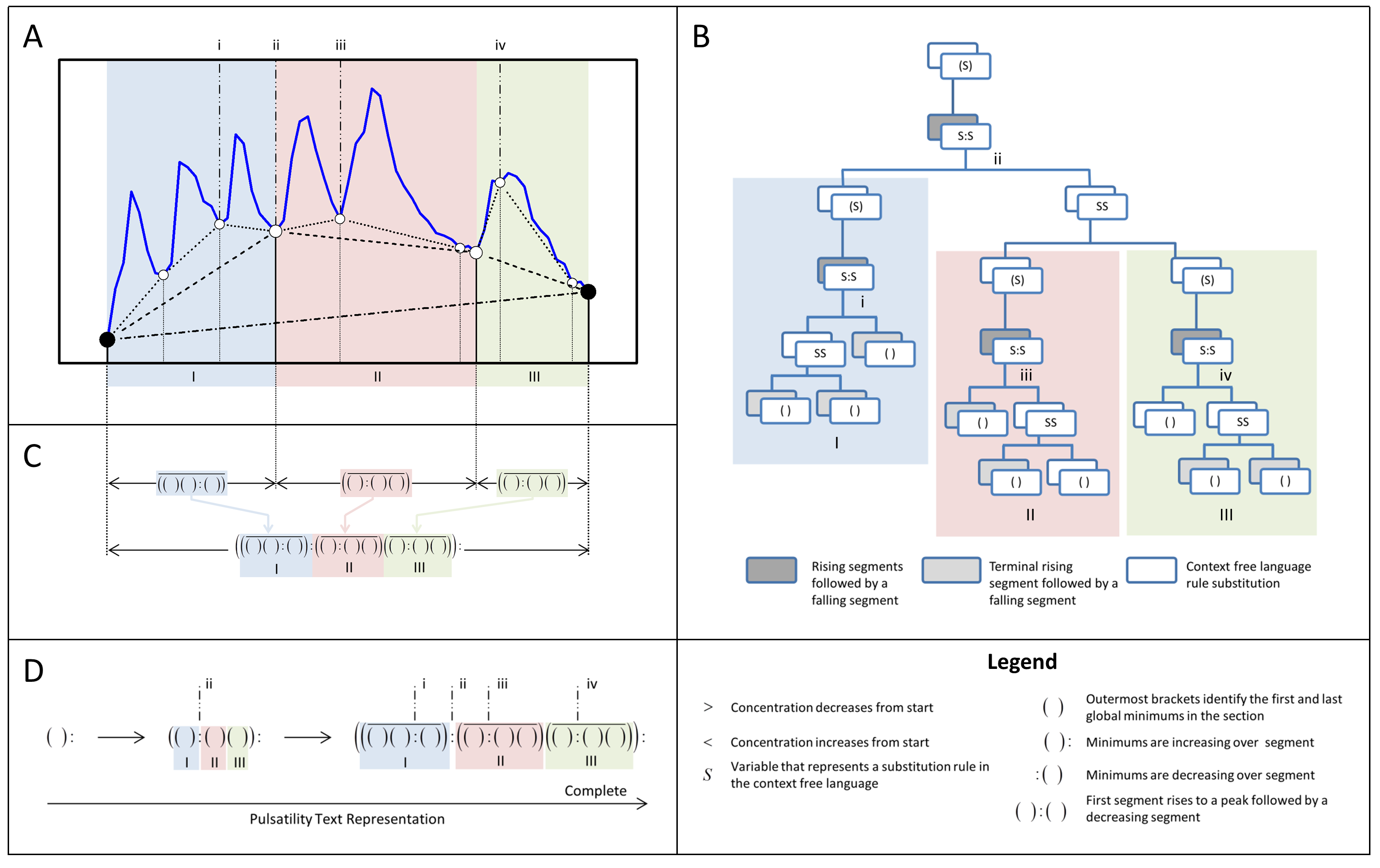

Supplement: Figure S5 — Linking time-series (data), the production graph and pulsicons. (TIF) [file pone.0104087.s005.tif]
