# Supplementary material for: Biological Time Series Analysis Using a Context Free Language: Applicability to Pulsatile Hormone Data
Source: PLoS One. 2014 Sep 3;9(9):e104087. doi: 10.1371/journal.pone.0104087 (PMC4153563; doi:10.1371/journal.pone.0104087)
Supplement: Text S1 — Supportive text and examples. (DOCX) [file pone.0104087.s006.docx]

**Supplemental Material**

# A. Context Free Languages

Context free languages (CFL) have been used to accomplish a variety of tasks including pattern matching [[1](#_ENREF_1)], cognitive data modeling [[2](#_ENREF_2)], cognitive development stage identification [[3](#_ENREF_3)], molecular query language processing [[4](#_ENREF_4)], and network hardware acceleration [[5](#_ENREF_5),[6](#_ENREF_6)]. The study of formal languages developed in conjunction with the design of early computers with the goal of determining the properties required to perform computations, to study convergence characteristics of algorithms, and to design programming languages for executing instructions on newly designed hardware [[7-9](#_ENREF_7)]. Currently, the study of formal languages is a requirement of most computer science departments with the goal of teaching abstract and mathematical computational reasoning [[10](#_ENREF_10),[11](#_ENREF_11)]. Formal language curriculum topics generally include aspects of regular languages and CFLs. Regular languages can be recognized with a finite state machine, and CFLs can be recognized with a pushdown automaton (PA). Both contain finite control (pre-defined transitions between states), but the PA includes a finite memory or pushdown stack equal in size to the input. No memory is required for a finite state machine. Neither language (regular or CFL) is as powerful as Turing recognizable languages, which include languages recognized by modern day computers. Thus, a CFL is capable of identifying more languages than a regular language; but, less languages than what can be identified with Turing recognizable languages. In this section, CFLs are presented using an example, with the goal of defining the key features required to discuss the application to hormone time-series analysis.

Rules for maintaining balanced parentheses are presented as an example of a CFL. Let $L_{P}$

be the set of strings containing valid parenthetical expressions. The CFL $L_{P}$ is composed of a grammar that produces valid strings in the language that can be transformed into a computation for recognizing the language. The context free grammar that produces parentheses is composed of a quadruple that includes the alphabet $V\to\left\{(,)\right\}$, the set of terminals $\Sigma\to\left\{(,)\right\}$, the set of rules $R\to\left\{S\to SS,S\to(S),S\to()\right\}$, and the start symbol $S\to\left\{S\right\}$. As an example, the valid expression parenthetical expression would require the following productions: $S\to\left(S\right)$, $\to\left(SS\right)$, , , , , and .

Determining whether a string is a member of a CFL requires a pushdown automata (PA). The formal definition of a PA includes (1) the set of states, (2) a string alphabet, (3) the start symbol, (4) the state transition rules, (5) a finite alphabet of the stack symbols, and (6) the transition function. Hence, a PA is referred to as a sextuple. A PA requires three key features: (1) the ability to remember the state or current position of computation, (2) a way to save portions of the string that requires additional information to process, and (3) a way to non-deterministically assess the appropriate state transition rule required to generate the string. The appropriate state transition required to generate a valid string is called a production. The grammar for generating parentheses, requirements for a PA, and a PA for recognizing a valid parenthetical expression are shown in Figure S1.

A tree (Figure S2-C) can also be used to visualize the productions required to produce a valid string in a language. The tree begins with a start symbol. Productions can then be added as necessary for each substitution. Consider the string which is a member of $L_{P}$. To test whether a string is valid it can be ‘processed’ by the PA described above. The string is read from right to left according to the rules of the PA with right parenthesis being added to the stack as encountered. As a left parenthesis is encountered, a right parenthesis is popped from the stack. The steps required to parse the string can be visualized as a tree, see Figure S2 for example.

Two issues required for developing an appropriate CFL include (i) the development of unambiguous grammars and (ii) insuring that there exists a valid production tree for every valid string in the language. Developing unambiguous grammars requires both conventions (e.g., processing strings from left to right) and carefully design of unique production rules. Insuring there is a valid production tree for every valid string in the language involves reasoning about the nondeterministic nature of a CFL. Consider the tree shown in Figure S2-C, from a computational perspective, it is not clear which production will result in successful termination; this makes the computation non-deterministic. From a practical perspective, a poorly defined CFL can result in an inefficient algorithm for finding a valid production or a production that does not converge appropriately. Despite these challenges, CFLs are used in many disciplines to support complex searches, to represent data in a hierarchal way, and to support bioinformatics applications.

# B. HAP Analysis Program

Overview. The HAP_Analysis Program is a tool developed to apply HAP analysis to a data set stored in a MATLAB structure. The graphical user interface includes sections for selecting data, for performing HAP analysis, and for application functions. The user can:

- Select a database of signals that are stored as a structure in a MATLAB binary file (See below for additional detail)
- Load the database to memory and display the signals
- Select a signal for analysis. This signal can be viewed for verification
- Perform HAP analysis on the selected signal or all the signals in the database; a single mouse click is required to start the analysis

Results and figures are generated automatically. Results are displayed in the console window as they are generated and figures are written to disk automatically. Functions for tiling and closing all windows are provided. A screen shot of the HAP_Analysis program with generated figures is shown in Figure S3 and is further described below. A demonstration of the program in use is available on the program’s GitHub page (<https://github.com/DennisDean/HapSource/releases>). Here, the analysis pipeline, output, and program requirements will be described. Several publically available routines are used in the program, and they are acknowledged below. The source code can be downloaded from <https://github.com/DennisDean/HapSource> and a version compiled for Windows can be found <https://github.com/DennisDean/HapSource/releases>.

Program Input. The program input is a data structure named ‘hormone_database’ that is stored as a MATLAB binary file. The input structure is used to populate menus that contain an entry for each subject. In addition, information required for labeling figures and creating tables is included. The structure’s fields are described in Table S1. The descriptive information within the input structure is required to effectively document the computation and for automatic reporting of HAP_Analysis program results. An example MATLAB script for creating the HAP_Analysis input structure can be found at https://github.com/DennisDean/CreateHapDB. The structure is easily extended to include additional information and can be tailored for other applications.

## Program Analysis Pipeline

The HAP_Analysis Program Pipeline is composed of five steps: (1) Visual Verification, (2) Feature Distribution Generation, (3) Feature-Feature Scatter Plot Generation, (4) HAP Summary Plot Generation, and (5) Pulsicon Generation. In addition, the HAP Analysis Pipeline is designed to provide intermediate results and to reduce the amount of time to analyze HAP output.

Visual Verification. The program first displays the input data. The peaks and nadirs identified at each recursive step of the algorithm are shown in a second plot, resulting in an animation of the algorithm. These figures are provided for the user to verify input and that the algorithm is working accordingly. Animation for a 24-hour cortisol profile is posted on the HAP project GitHub page (<https://github.com/DennisDean/HapSource/releases>).

Feature Distribution Generation. Histograms of the features (e.g. rise time, rise amplitude, and accumulation) extracted at each recursion levels are created. The figures are included to provide the user with intermediate analysis results.

Feature-Feature Scatter Plot Generation. Scatter plots of extracted features are provided to allow the user to quickly identify potential relationships in the extracted features. Scatter plots are generated for both raw features (e.g. rise time) and computed features (e.g. accumulation).

HAP Summary Plot Generation. HAP summary plots are provided as a way to compactly review the HAP analysis results. Accumulation, dissipation, and inter-nadir intervals versus recursion number are plotted separately. An accumulation versus dissipation plot is also generated.

Pulsicon Generation. Pulsicons are generated at each analysis iteration. Note that both a text and a LaTex version of the pulsicon are created. The text version is used for console displays, and the LatTex version is used for figure generation.

In order to further clarify the output generated during the pipeline analysis, a complete summary of figures generated for a single 24 hour cortisol profile is provided in Table S2. In addition, a video illustrating the generation of these figures is available on the projects GitHub page (<https://github.com/DennisDean/HapSource/releases>).

Requirements and Installation. The MATLAB source code can be found on the HAP GitHub page (https://github.com/DennisDean/HapSource). A compiled version of the program is provided as Microsoft Windows executable. The application requires the MATLAB Compiler Runtime (MCR) which enables machines to execute MATLAB code without having MATALAB stored on the machine. The MCR can be downloaded from the MATLAB website (<http://www.mathworks.com/products/compiler/mcr/>). The MCR does not need to be installed if MATALB if MATALB is installed on the computer. The Windows executable was generated with MATALB 2013a. The compiled version of the program can be found on the HAP GitHub page (https://github.com/DennisDean/HapSource/releases).

The program is written in MATLAB which is widely available to researchers. In order to create the required input structure, a user needs to be proficient in MATLAB file reading and writing commands. Future work can create an interface for non-MATLAB-proficient users. The analysis automatically generates results that are provided to the user through the MATLAB console and graphically through the generation of figures during the data analysis process. Results written to the console and generated figures are stored automatically to disk. The MATLAB script files are versioned ([www.github.com](http://www.github.com)), which allow tracking of changes and forms the basis for modifications and extensions. Individuals interested in modifying or extending the code base should contact the lead author for access to script files, which are accessible from the lead author’s GitHub account (http:www.github.com/DennisDean). Interacting via the GitHub account will allow for bug fixes to be tracked and derived enhancement to be integrated within the existing code base.

Window tiling functions use code available from MATLAB Central file exchange area, which is a MATLAB sponsored code repository. Initially, tilefigs.m (<http://www.mathworks.com/matlabcentral/fileexchange/>38581-tilefigs) was used. Currently, tilefigs from the Figure Management Utilities is used (<http://www.mathworks.com/> matlabcentral/fileexchange/12607-figure-management-utilities). The file tiling code is made available under a BSD license, which allows redistribution of source code and binary form with the condition that the copyright and disclaimer are included. The ‘Latex Figure Output’ was used to create figures from the LATEX strings generated by HAP (<http://www.mathworks.com/matlabcentral/fileexchange/13531-latex-figure-output/content/latex_figure.m>). The scattermatrix function from the data visualization toolbox (www.datatool.com) was used to create scatter matrices.

# C. Comparator Operators

The family of comparator operators systematically identifies features of the data. These data features are best understood by relating the operators to a visual interpretation of the data. Two quantitative comparators, each from the set $\left\{<,>,=\right\}$ are used to identify data features. The three comparators results in a 3 by 3 matrix of operators (Figure S4 C, D, E). The operators along the diagonal correspond visually to Peaks (P), Nadirs (N), and Flat (F) regions in the data. Operators at matrix position (1,2) and (2,1) correspond to the Decreasing (D) and Rising (R) operators. Operators that correspond to transitioning to or from a flat regions are defined at positions (1,3)(Decreasing to Flat), (2,3)(Rising to Flat), (3,1)(Flat to Rising), and (3,2)(Flat to Decreasing). The collection of operators corresponds to the set of identifiable features in the data. Hierarchical data features can now be defined.

# D. Relationship between Production Graph and Pulsicon (Detailed Example)

A detailed example demonstrating the relationship between production graphs and pulsicons for a segment of cortisol data is presented.

## Production Graph

The CFL production graph is presented as a novel visualization tool for describing the qualitative features present in a cortisol time-series. The CFL production graph is a summary of the steps in the language required to represent the data. The goal of the graph is to better visualize the relations of embedded pulses (qualitative features) and to produce a rigorous framework for future analytical work.

Details of one individual cortisol time-series production graph are shown in Figure S5, which contains an approximately 10 hour time-series segment (Figure S5A). The production graph (Figure S5B) is a summary of the steps required to generate the pulsicon (Figure S5C) and is presented as a means to visually inspect qualitative differences in the pulsatility. The production graph for the subject is generated according to the language rules. Productions that generate a single subsequent production are shown with a vertical line $\left(\left\{S,\left(S\right)\right\}\right)$. The remaining rules generate $\left\{SS,S:S\right\}$, two productions that are represented with a left and right branch. In this example, the ‘S’, ‘:’ and ‘S’ symbols respectively represent the rising portion, the peak, and falling portion of the data. Points in time and temporal intervals are identified in Figure S5 to reinforce the relationship between the time series, the pulsicon, and the production graph. Specifically, the dotted vertical lines with lower case roman numerals represent the same time points, and the shaded rectangles labeled with uppercase roman numerals represent the same temporal interval.

## Pulsicons

Figure 5C illustrates the pulsicons for the 10-hour data segment of Figure 5A. The hierarchical nature of pulsicons allows for rises and falls at different recursive steps to be shown. The recursive nature of the pulsicon allows for different levels of detail to be displayed as shown in Figure 5D.

# E. Nadir Selection Algorithm Implementation: Core Analysis Routines

The Nadir Selection Algorithm is the core data analysis routine that drives the computations described in the Methods section. The algorithm is implemented in MATLAB as a pair of interacting script files. The calling script file (pulseSegmentation.m) creates an abstract view of the time-series from which production graphs and pulsicons are derived. A lower level script file (getPeaks.m) implements the comparator operator (See **Supplementary Materials** section C). Both files are implemented as MATLAB classes and contain approximately 4,500 lines of code.

The pulseSegmentation class recursively passes time-series sections to the getPeaks class and determines the hierarchical organization of the getPeaks output. At each recursive step, the pulseSegmentation class identifies data sections for which there are sufficient nadirs and peaks and at least one rise and fall exists. Each identified data section is passed to a new getPeaks instantiation. The pulseSegmentation class terminates recursive calls to the getPeaks class when there are no rises and falls in the data sections identified during the analysis.

The getPeaks Class is a low level analysis function that identifies local peaks and nadirs within a data section by applying the matrix of comparison operators. Application of the comparison operators results in a list of descriptive interpretations of the data section (e.g., peak, nadir, and decreasing to flat). A data structure that includes the information about the identified peaks and nadirs as well as the interpretation is returned to the pulseSegmentation class.

Upon termination of the pulseSegmentation class, a segmentation structure is created and contains sufficient information to generate production graphs and pulsicons. The source code contains additional details regarding the content and structure of the segmentation structure (See Software subsection of the Methods for information on how to access the software).

Both the pulseSegmentation and getPeaks class include functions for reporting intermediate and final status results. These classes are configured to echo both intermediate and final results to the MATLAB console. The pulseSegmentation class includes functions for overlaying intermediate results on the input data, which can be used to create plots at any step in the recursive algorithm. The same functions used for overlaying results on the data can also be used to create data animations.

# F. Mathematical Model of Cortisol Pulsatility Description

The stochastic differential equation model of cortisol concentrations described in Brown et al. was used to randomly generate 24-hour cortisol profiles [[12](#_ENREF_12)]. The cortisol model is designed to simulate realistic cortisol time-series with parameters represented as distributions. Model parameters were defined by fitting distributions to published data. The interpulse interval is modeled as a gamma distribution. The circadian amplitude modulation is modeled as a two harmonic sinusoid with amplitude parameters that co-vary with each other. The period of the circadian concentration amplitude modulation is assumed to be 24 hours. Twenty-four hour cortisol concentration profiles were simulated by (1) sampling the interpulse intervals for the 24-hour period, (2) sampling the circadian modulation parameters [12], and (3) setting the clearance parameter (gamma) to parameters set in [12]. The MATLAB script files used to generate the simulated data and simulated data can be found at <https://github.com/DennisDean/HapBrownCortisolModel>. The cortisol simulation MATLAB code was adapted from a cortisol model implementation developed by David Nguyen. Dr. Nguyen developed the code as part of a reversible jump Monte Carlo project for which he developed analytics and informatics [[13](#_ENREF_13)].

**References**

1. Phaninadra G, Shankar KR, Sreenivas PD (2007) A Fast Multiple Matching Algorithmn using Context Free Gammar and Tree Model. International Journal of Computer Science and Network Security 7: 231-234.

2. Purdy BP, Batchelder WH (2009) A context-free language for binary multinomial processing tree models. Journal of Mathematical Psychology 53: 547-561.

3. Commons ML (1998) Hierarchical Complexity of Tasks hows the Existance of Developmental Stages. Developmental Review 18: 237-278.

4. Proschak E, Egner JK, Chler A, Chneider G, Echner U (2010) Molecular Query Language (MGL) - A Context-Free Grammar for Substructure Matching. Journal of Chemical Information and Modeling 47: 295-301.

5. Moscola JM (2008) Washington University: Washington University. 1-162 p.

6. Moscola J, Cho YH, Lockwood JW. Context-Free Gammar Parsing for High-Speed Network Applications in Reconfigurable Hardware 2005.

7. Pullum G, Gazdar G (1982) Natural languages and context-free languages. Linguistics and Philosophy 4: 471-504.

8. Chomsky N (1956) Three models for the description of language. IRE Transaction on Information Theory 2: 113-124.

9. Knuth DE (1968) Semantics of Context-Free Languages. Mathematical Systems Theory 2: 127-145.

10. Sipser M (1997) Introduction to the Theory of Computation: Course Technology. 1-416 p.

11. Hopcroff JE, Ullman JD (2006) Introduction to automata theory, Languages, and Computation. Massachusetts: Addison-Wesley. 1 p.

12. Brown EN, Meehan PM, Demster AP (2001) A stochastic differential equation model of diurnal cortisol patterns. American Journal of Physiology - Endocrinology and Metabolism 280: E450-E461.

13. Nguyen DP, Frank LM, Brown EN (2003) An application of reversible-jump Markov chain Monte Carlo to spike classification of multi-unit extracellular recordings. Network: Computation in Neural Systems 14: 61-82.

**Tables**

**Table S1:** Fields description for HAP_Analysis input structure

| **Description** | **Data description** |
| --- | --- |
| Subject_info | A data structure that includes subject and group |
| Subject_data | Subject data is stored in a cell array |
| T | Duration (minutes) of the data to analyze from the start of the time-series |
| delta_t | Data sampling interval (minutes) |
| Conditions | Cell array of conditions. This is currently limited to scheduled sleep and wake conditions |
| Groups | Group labels are stored in a cell array, one entry for each group identified in the subject info structure |
| num_subjects | The number of subjects included in the dataset |
| units | The time-series data units |

**Table S2:** Fields description for HAP_Analysis Program Data Structure

| **Figure** | **Figure Type** | **Description** |
| --- | --- | --- |
| 1 | Partitioning | Data partitioned in to hierarchical rise and falls |
| 2 | Histogram | Histogram of rise duration |
| 3 | Histogram | Histogram of amplitudes |
| 4 | Histogram | Histogram of fall duration |
| 5 | Histogram | Histogram of amplitude descent |
| 6 | Histogram | Histogram of inter-nadir interval |
| 7 | Amplitude vs. Duration | Rise amplitude vs. duration |
| 8 | Amplitude vs. Duration | Descent amplitude vs. duration |
| 9 | Amplitude vs. Duration | Rise and descent amplitude vs. duration with linear fit |
| 10 | Histogram | Histogram of accumulation rate |
| 11 | Histogram | Data histogram of dissipation rate |
| 12 | Scatter Plot Matrix | First iteration rise times, amplitude, fall times and amplitude falls scatter matrix |
| 13 | Scatter Plot Matrix | Second iteration rise times, amplitude, fall times and amplitude falls scatter matrix |
| 14 | Recursion Plot | Accumulation rates by recursion number. Time since the start of the time series ins identified by color. |
| 15 | Recursion Plot | Dissipation rates by recursion number. Time since the start of the time series is identified by color. |
| 16 | Recursion Plot | Interpulse interval by recursion number. Time since the start of the time series is identified by color. |
| 17 | Dissipation vs. Accumulation | Dissipation vs. accumulation plotted on a log-log scale. Data points identified by recursion number (shape) and location along the time (thirds, color) |
| 18 | ScatterPlot Matrix | First iteration accumulation, dissipation and inter-nadir interval scatter matrix |
| 19 | Scatter Plot Matrix | Second iteration accumulation, dissipation and inter-nadir interval scatter matrix |
| 20 | Pulsicon | Characterize the initial and final portion of the signal |

**Figures**

**Figure S1:** Specification of a CFL for recognizing parenthesis.

(A) A context free grammar or language for generating a valid parenthetical string is presented. This grammar is specified as a 4-tuple with the alphabet $V$ defined as , the set of terminals $\Sigma$ defined as , the set of production rules $R$ defined as , and the start symbol $S$ defined as $\left\{S\right\}$. (B) The key features of a pushdown automata are shown graphically: 1) an input string shown with input start (*) and end symbol (&), (2) finite control which includes a movable read head, and (3) a pushdown stack. (C) A pushdown automata that recognizes valid parenthetical expressions is shown. Each letter of the input string is read at state p. when a ‘)’ is read a ‘)’ is pushed on the stack which is summarized as ‘’. When an ‘(‘ character is read a ‘)’ parenthesis is popped from the stack which is summarized as ‘$\begin{matrix} ( & , & ) & / & \varepsilon\end{matrix}$’. If the string is processed and the stack is empty, the state Q is reachable and the input string is valid.

**Figure S2:** Processing of a string of parenthesis with a CFL.

(A) A syntactically correct parenthetical string. (B) The step by step processing of the example string by the pushdown automata in (A) is shown. The state, unread input, and the stack are listed at the start and after the processing of each character. Processing of each character occurs at state P. State Q represents the quit or successful termination state. The $\varepsilon$ characters in the last line in the table represent an empty input string and an empty stack, respectively. (C) A parse tree representations of the production rules that could generate the example string. The parse tree is produced top down, resulting in a graph of productions with each tree path ending in a terminal character. The input string can be reproduced by substituting the terminal characters from the bottom of the tree up to the start symbol.

**Figure S3:** HAP_Analysis output.

HAP_Analysis Program’s Graphical User Interface (GUI) and a subset of figures generated by the program. (A) GUI. HAP_Analysis Program’s GUI for initiating the Program is shown. (B) Visual Verification. Raw data and summary of HAP analysis steps. During execution, the recursive HAP steps are animated in the second figure of Panel B. (C) Feature Distribution Generation. HAP extracted features including accumulation, dissipation, and inter-nadir interval are generated. (D) Feature-Feature Scatter Plot Generation. Scatterplot matrix of HAP extracted features and parameters are created. (E) HAP Summary Plot Generation. The HAP summary plots visualize the HAP_Analysis results. Accumulation and dissipation versus the HAP recursion number are plotted separately along with a figure that plots dissipation versus accumulation (F) Pulsicon Generation. A pulsicon is generated recursively and results in a text description of the data.

**Figure S4:** Definition and interpretation of the comparator operator.

(A) The comparator operator is a function of three consecutive data points and a pair of numerical comparators. The comparator operator characterizes the relationship between the three points with a pair of Boolean operators relative to the middle point. For example, (>,>) means that the second point is greater than both the first and third points. (B) Each operator in the pair is a member of the operator set that includes a less than (<), greater than (>), and equal operator (=). All possible pairs of comparison operators are shown in a two dimensional grid. (C) A text description of the pairs of operators shown in B. (D) Shorthand text for the comparator operators in B and described in C. (E) The visual interpretation of the enumerated comparator operators presented in B.

**Figure S5:** Linking time-series (data), the production graph and pulsicons.

The dotted vertical lines with lower case roman numerals (i, ii, iii and iv) represent the same time points in panels A, B, C, and D. The uppercase roman numerals (I, II, and III) represent the same temporal intervals in panels A, B, and C. (A) The time-series graph with pulsicons associated with temporal intervals. (B) The production graph. (C) Pulsicons are generated at each step of the algorithm and represent specific temporal intervals within the time-series. (D) Pulsicons from different analysis iterations for this data set. (Legend) is for symbols in panels B, C, and D.
